# Unique Halloysite Nanotubes–Polyvinyl Alcohol–Polyvinylpyrrolidone Composite Complemented with Physico–Chemical Characterization

**DOI:** 10.3390/polym9060207

**Published:** 2017-06-06

**Authors:** Tayser Sumer Gaaz, Abdul Amir H. Kadhum, Patina Kiah Anak Michael, Ahmed A. Al-Amiery, Abu Bakar Sulong, Mohamed H. Nassir, Ahed Hameed Jaaz

**Affiliations:** 1 Department of Mechanical & Materials Engineering, Faculty of Engineering & Built Environment, University Kebangsaan Malaysia, Bangi, Selangor 43600, Malaysia; abubakar@ukm.edu.my; 2 Department of Machinery Equipment Engineering Techniques, Technical College Al-Musaib, Al-Furat Al-Awsat Technical University, Al-Musaib, Babil 51009, Iraq; 3 Department of Chemical & Process Engineering, Faculty of Engineering & Built Environment, Universiti Kebangsaan Malaysia, Bangi, Selangor 43600, Malaysia; amir8@ukm.edu.my (A.A.H.K.); caddy.tina@gmail.com (P.K.A.M.); 4 Energy and Renewable Energies Technology Centre, University of Technology, Baghdad 10001, Iraq; 5 Program of Chemical Engineering, Taylor’s University-Lakeside Campus, Subang Jaya, Selangor 47500, Malaysia; mhnassir1949@gmail.com; 6 Solar Energy Research Institute (SERI), Universiti Kebangsaan Malaysia, Bangi, Selangor 43600, Malaysia; eng_tay83@yahoo.com

**Keywords:** Halloysite nanotubes, polyvinyl alcohol, polyvinylpyrrolidone, physico–chemical properties, surface area

## Abstract

A halloysite nanotubes–polyvinyl alcohol–polyvinylpyrrolidone (HNTs–PVA–PVP) composite has been investigated for a quite long time aiming at improving the physico–chemical characterization of HNTs. In this work, HNTs–PVA–PVP composite were prepared based on a unique procedure characterized by crosslinking two polymers with HNTs. The composite of two polymers were modified by treating HNTs with phosphoric acid (H_3_PO_4_) and by using malonic acid (MA) as a crosslinker. The composite was also treated by adding the dispersion agent sodium dodecyl sulfate (SDS). The HNTs–PVA–PVP composite shows better characteristics regarding agglomeration when HNTs is treated in advance by H_3_PO_4_. Fourier transform infrared spectroscopy (FTIR), X-ray diffraction (XRD), transmission electron microscopy (TEM), field emission scanning electron microscopy (FESEM), brunauer–emmett–teller (BET), size distribution, and atomic force microscopy (AFM) are used to characterize the physio-chemical properties of the composite. FTIR shows additional peaks at 2924.29, 1455.7, and 682.4 cm^−1^ compared to the neat HNTs due to adding MA. Despite that, the XRD spectra do not show a significant difference, the decrease in peak intensity could be attributed to the addition of semi-crystalline PVA and the amorphous PVP. The images taken by TEM and FESEM show the possible effects of MA on the morphology and internal feature of HNTs–PVA–PVP composite treated by MA by showing the deformation of the matrix. The BET surface area increased to 121.1 m^2^/g compared to the neat HNTs at 59.1 m^2^/g. This result, the second highest recorded result, is considered a breakthrough in enhancing the properties of HNTs–PVA–PVP composite, and treatment by MA crosslinking may attribute to the size and the number of the pores. The results from these techniques clearly showed that a significant change has occurred for treated HNTs–PVA–PVP composite where MA was added. The characterization of HNTs–PVA–PVP composite with and without treating HNTs and using crosslinker may lead to a better understanding of this new composites as a precursor to possible applications in the dentistry field.

## 1. Introduction

Halloysite, similar to kaolinite, is a natural clay mineral characterized by a nanotubular structure whose chemical formula is expressed as Al_2_Si_2_O_5_(OH)_4_·*n*H_2_O [1,2,3,4,5]. HNTs have an external diameter of 30 to 190 nm and an internal diameter of approximately 10 to 100 nm [2]. There are several reasons for such nano dimensions to be of great interest. HNTs are unique, versatile and consist mainly of aluminosilicate nanoclay. HNTs are eco-friendly nanotubes with lower cost than carbon nanotubes (CNTs). HNTs have numerous applications owing to their excellent characteristics such as additives in polymers and plastics, electronic components, thermoplastics, drug delivery, vehicles, cosmetics and biomedical applications [6,7,8,9,10]. Although such composites have good thermal properties, they are not biodegradable. Generally, the natural nanotube materials can disperse due to their non-uniform structure. The physico–chemical properties are affected by the mismatch of HNTs tetrahedral layer and the octahedral layer which can be detected by intercalation in addition to the capacity of cation exchange [11]. When clay materials are considered as minerals, their main use is in the field of high-quality porcelain [12]. Another property of HNTs is the ability to be added polymers as additives. Moreover, the hollow structure of HNTs gives them potential as supporters in medical fields including drug delivery, enzymes and many others [11]. Through the dispersion of natural nanotube structural clays, it is convenient to obtain uniform nanotube structure for wider applications [13]. It was found that even small loadings of HNTs were sufficient to make significant changes in the polymer properties [14]. In particular, the surface morphology is very sensitive to the nature of the polymer [8].

Several methods have been used and tried to improve the surface properties of clay minerals including HNTs in particular. These improvements include mechanical properties [9,15], intercalation [16], thermal properties [17], and chemical activation [8,18]. For chemical activation, various acids were utilized such as hydrochloric acid [18], sulfuric acid [19], H_3_PO_4_ [20], and pectin [9,21]. Generally speaking, acids cause elimination of impurities, dissolution of the external layers, disaggregation of nano particles, and, most importantly, destruction of the structure which leads to a better surface activity by increasing surface area and pore number and/or volume [18]. H_3_PO_4_ is the least used acid found in the literature. Consequently, in this article, H_3_PO_4_ was used for HNTs treatment in order to shed the light on its effects on the physical and chemical properties of the HNTs and to compare these effects with the effects of other acids.

The process of chaining two polymers together via either covalent or ionic bond is called crosslink. In synthetic polymer field, in particular, the crosslink could change the physical and chemical properties. Modifying part or all of these properties owes the crosslink that is widely used as a promoter in chemistry and biological sciences with the extent that varies from polymer to another. In nano field, the dicarboxylic acid is known as malonic acid (MA), whose structure is CH_2_(COOH)_2_, plays an important role as a competitive inhibitor [22]. For these reasons, MA is used as a crosslinker with minimal toxicity compared to glutaraldehyde or glyoxal [20]. MA is then used to build the block of chemicals in order to produce numerous valuable compounds [23] which are, generally, biodegradable thermoplastics that are commonly used in foam packaging, bags, and other related products. The mechanism of using MA as a crosslinker was described by Qiu and Netravali [12]. When individual HNTs are mixed with polyvinyl alcohol (PVA), the HNTs–PVA is characterized as a fully biodegradable composite [12]. The most important step in this mechanism is producing stable individualized HNTs crosslinked with PVA using MA and acid as a catalyst. MA-carboxylic groups (COOH) react with the hydroxyl groups (OH) in the PVA to form ester linkages. The product, HNTs–crosslink–PVA, is a water-insoluble which makes these products eligible for commercial applications. The HNTs–crosslink–PVA composites are also characterized as having excellent physical properties.

Thermoplastic polymers such as PVA are also biocompatible polymers. PVA, in particular, has been widely used in industry for films, paper, and adhesive coating for its attractive properties. In addition, PVA is soluble in water due to hydrophilic properties where the hydroxyl [–OH] group is bonded to alternating carbon atoms and, as such, it helps to promote its degradation through hydrolysis [24,25]. The HNTs–PVA composite, on the other hand, was found earlier without proper HNTs individualization [26]. The present work attempts to individualize HNTs by incorporating MA with PVA in order to achieve better qualities of the composite. Generally, SDS has been used to improve the dispersion ability of nanomaterials by forming an aqueous solution [27]. Briefly, esterification is obtained via HNTs–crosslinked–PVA [28,29]. Researchers have been exploring different levels of how MA crosslinking is effective and, consequently, their expectations varied within a reasonable level of differences especially concerning thermal properties. The highest level of PVA–MA crosslinking was currently achieved by producing water-insoluble material. In addition, PVA–MA crosslinking has produced smooth surfaces that play an important role in enhancing the physical properties of HNTs–crosslinked–PVA composite [30]. The MA crosslinked HNTs–PVA composite showed even better properties for medicinal and bio-medicinal uses [12,31].

Polyvinylpyrrolidone (PVP) is one of the commonly used polymers in medical applications due to its water solubility and very low cytotoxicity. Additionally, when PVP is considered as a matrix or an additive, it can be used as a controlled agent for drug release for the co-precipitation of other drugs and as a solid dispersion for controlling drug diffusion. The combination of the properties of PVA and PVP in PVA–PVP blends has emerged as a new tool for the preparation of biomaterials [32]. PVP has a good reputation due to its outstanding absorption and complexes abilities [33]. PVA and PVP are known to form a thermodynamically miscible pair and improve the mechanical properties of PVA in PVA–PVP [32,34]. PVP is used as polymer dispersing agent, as it will optimize the dispersion by wrapping the surface of nanotube [35].

The objective of this article is to investigate the properties of HNTs mixed with PVA and PVP. This research also investigates the morphological, physical and chemical properties of the HNTs–PVA–PVP composite before and after crosslinking with MA and functionalized HNTs.

## 2. Materials and Methods

### 2.1. Materials

Natural Nano, Inc. (832 Emerson Street Rochester, New York, NY, USA), supplied HNTs. Table 1 and Table 2 contain the chemical composition and physical properties of HNTs, respectively. Other chemicals used in this work and their properties are presented in Table 3.

### 2.2. Procedures of Composite

Table 4 explains the composition of the samples used in this article. The neat HNTs are named S01. S01 was used to prepare the second sample, S02, by adding 15 g of S01 to 100 mL of 3 M H_3_PO_4_. The solution is kept in a water bath at a steady temperature of 90 °C while stirring process was performed at speed of 200 rpm for 3 h. The mixture is centrifuged at a speed of 3000 rpm for 10 min to separate the paste from the solution. The paste was washed away using distilled water up to four times until the morality of pH 7 is achieved and then dried in an oven at 70 °C for 12 h. The solid sample (S02) was taken from the oven and ground and kept aside for other uses. S01 and S02 were used separately to prepare four other samples. The process of preparation of these four samples is similar, where PVA and PVP are mixed together first and then mixed with neat HNTs and treated HNTs to produce S03 and S04, respectively. The other two samples are prepared by adding a mixture of PVA, PVP, and the crosslinker MA to S01 and S02 to produce S05 and S06, respectively. The mixture of PVA and PVP or PVA, PVP, and MA was added to the dispersive agent SDS and stirred at 500 rpm for 1 h at 90 °C.

### 2.3. Characterization Techniques

In this section, the characterization of S01 and S02 are presented by utilizing FTIR, XRD, TEM, FESEM, BET, Zeta Potential, Size Distribution, and AFM. Firstly, FTIR is used to identify the functional group of S01. The analysis is performed using a Perkin Elmer System 2000 (Waltham, MA, USA), which is equipped with attenuated total reflectance. For this research, FTIR of resolution4 cm^−1^ was run between 600 and 4000 cm^−1^. The structure and the size of HNTs crystal were investigated by XRD (Bruker Corporation, Berlin, Germany, D8 with AXS X-ray Bruker and Cu tip of wavelength of 1.5406 Å). This model is supplied with EVA-V2 software (Bruker Corporation, Karsruhe, Germany). The patterns are compared with the standard patterns issued by the Joint Committee on Powder Diffraction Standards (JCPDS). HNTs was also investigated by TEM (Philips, model CM12 (Somerset, NJ, USA) for morphological images because it is an essential test commonly used for morphology and particle size. This TEM model is operated at 80 kV which generates images through which the surface and the particle size can be studied. Preparing samples for TEM requires dispersing a proper amount in 10 mL ethanol where the solution is thoroughly mixed using watered-bath ultrasonic for 10 min. Besides TEM, FESEM (Zeiss SUPRA 55-VP, a product of Konigsallee, Düsseldorf, Germany) of high resolution and a low charging effect is used as supporter test for HNTs morphology. FESEM magnification was set at 25,000× and 50,000×. The elemental analysis of HNTs is investigated by EDS (OXFORD, Version 2, Dallas, TX, USA). The surface properties such as the surface area are investigated by BET (Gemini apparatus, ASAP 2020, micrometrics, Norcross, GA, USA) of an accuracy of ±0.02 m^2^/g. In order to perform BET, degassing samples should be conducted in a vacuum of 50 mTorr at a certain temperature (350 °C) for a period of 2 h. The software used for BET is Barrett–Joyner–Halenda (BJH) which is used to calculate the pore volume and the average pore size. The total surface area can be determined by desorption–adsorption of nitrogen. Malvern (Zetasizer nano zs) particle size analyzer, based on laser scattering method, the measured size distribution of HNTs. In order to measure particle size and distribution, the HNTs were suspended in distilled water and ultrasonication was used to breakup agglomerates and disperse the HNTs in water uniformly. Then, the mixture was charged in the testing chamber of particle size analyzer. After that, particle size distribution was calculated from the angle and intensity of the scattering beams. For analyzing the surface morphology and roughness of the membranes, atomic force microscopy was employed using the AFM apparatus (DI Nanoscope IIIa, Veeco, New York, NY, USA). The membrane surfaces were examined in a scan size of 10 µm × 10 µm.

## 3. Results and discussion

### 3.1. Fourier Transform Infrared Spectroscopy (FTIR)

**Figure 1:**
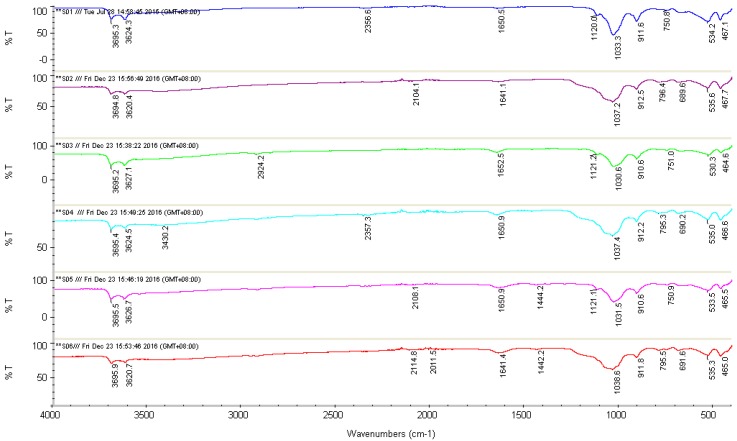
FTIR spectra of: (**a**) S01; (**b**) S02; (**c**) S03; (**d**) S04; (**e**) S05; and (**f**) S06.

Table 5 shows the wavenumbers and assignments of major IR vibration bands of FTIR spectra taken from Figure 1. The spectra of [O–H] stretching of the inner-surface hydroxyl of S01, S02, S03, S04, S05, and S06 show similar absorption bands at 3695.3, 3694.8, 3695.2, 3695.4, 3695.5 and 3695.9 cm^−1^, respectively. The presence of similar bands at 3624.3, 3620.4, 3627.1, 3624.5, 3626.7 and 3620.7 cm^−1^ show the [O–H] stretching of the inner hydroxyl group. The absorption band at 3430.2 cm^−1^ in S04 indicates the presence of OH stretching vibration due to the strong intramolecular hydrogen bonding [20]. The weak absorption band at 2924.2 cm^−1^ is assigned to –CH_2_ asymmetric stretching vibrations [36,37,38,39]. However, absorption bands observed between 2011.5 and 2357.3 cm^−1^ are due to the stretching of C–H bonds [20]. Absorption bands in S05 at 1444.2 cm^−1^ and 1442.2 cm^−1^ in S06 indicate the presence of C=O due to the crosslinking with MA [20]. Absorption bands for S01 and S02 recorded at 1650.5 cm^−1^ and 1641.1 cm^−1^, respectively, show very weak peaks that could not be reliably assigned to any possible bonds and might be due to impurities [40]. However, recorded bands for S03, S05, S04, and S06 at 1652.5, 1650.9, 1650.9 and 1641.4 cm^−1^, respectively, belong to the C=O stretching from amide group of PVP [37,38]. The bands at 1120.0, 1121.2 and 1121.1 cm^−1^ belong to Si–OH groups in S01, S03, and S05, respectively [40,41]. However, there is an absence of Si–OH groups for S02, S04, and S06 due to the influence of H_3_PO_4_ treatment on S01 [40]. The bands at 1033.3, 1037.2, 1030.6, 1037.4, 1031.5 and 1038.6 cm^−1^ for S01, S02, S03, S04, S05, and S06 were attributed to the vibration Si–O–Si group, which is closer to the surface of HNTs molecules [40,41,42]. Absorption bands at 911.6, 912.5, 910.6, 912.2, 910.6 and 911.8 cm^−1^ assigned to the Al–OH vibrations [40]. The bands at 750.8, 796.4, 751.0, 795.3, 750.9 and 795.5 cm^−1^ for S01, S02, S03, S04, S05, and S06 indicate the stretching and bending of Al–O–OH [40]. The bands at 689.6, 690.2 and 691.6 cm^−1^ for S02, S04, and S06 show the stretching mode of apical alcohol-OH out of plane bend [40].

### 3.2. X-ray Diffraction (XRD)

**Figure 2:**
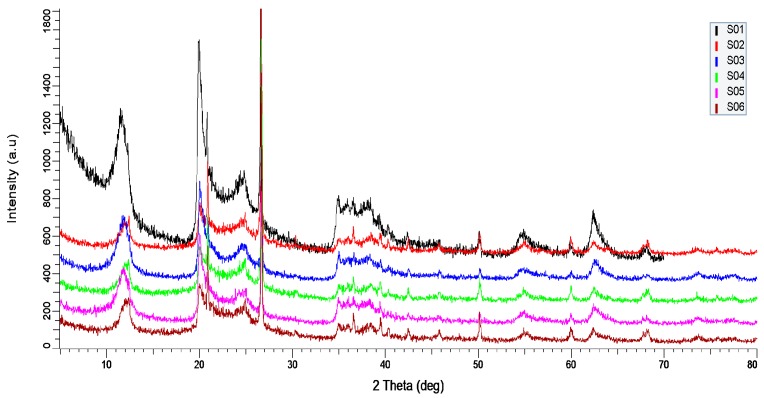
XRD patterns of (**a**) S01; (**b**) S02; (**c**) S03; (**d**) S04; (**e**) S05; and (**f**) S06.

XRD is a common non-destructive technique to study the crystallographic structure of materials. Figure 2 shows the XRD spectra of S01, S02, S03, S04, S05, and S06. As shown in Figure 2, the diffraction angle (2θ) was taken up to 70°. S01 shows sharp peaks at 2θ of 12.80°, 20° and 25° indicate the crystalline and tubular halloysite structure of HNTs [39,43]. However, it is observed that the peak intensity decreases but not significantly, due to the addition of PVA and PVP. This is because PVA is semi-crystalline polymer while PVP is amorphous [36,44]. The peak at *2*θ of 62.10° indicates the halloysite is dioctahedral mineral and at peak 2θ of 24.59° and 26.45° show the presence of silica in form of cristobalite and quartz [44]. As the result confirmed that although with the addition of semi-crystalline PVA and amorphous PVP, the crystallinity of HNT does not change.

### 3.3. Transmission Electron Microscopy (TEM)

**Figure 3:**
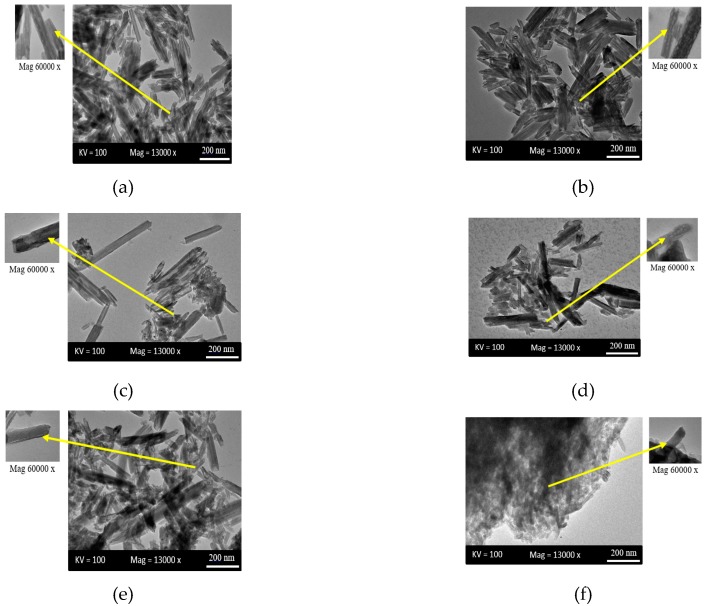
TEM images of low and high magnification of: (**a**) S01; (**b**) S02; (**c**) S03; (**d**) S04; (**e**) S05; and (**f**) S06.

The TEM images in Figure 3 show that the halloysite has a cylindrical shape and has a transparent central area that runs along the cylinder, indicating that nanotubular particles are hollow and open-ended. It has 200–1000 nm length, 10–50 nm inner diameter, and 80–150 nm outer diameter [40]. Figure 3b–e shows that the nanotubes started to scatter and individualized with noticeable agglomeration. These indicate a better dispersion due to the addition of polymers (PVA and PVP) and the dispersing agent SDS. The high viscosity of PVA helped individualized HNTs to prevent clustering by immobilizing them [20]. Dispersing agent SDS also reduces the agglomeration [13]. However, it can be observed that the outer surface of nanotubes in Figure 3b,e,f are exfoliated [40]. The nanotubes lost their physical appearance in Figure 3f due to severe exfoliation. The interaction between polymer molecule and clay layer increase in the presence of PVP which causes the formation of exfoliated composite [38].

### 3.4. Field Emission Scanning Electron Microscopy (FESEM)

**Figure 4:**
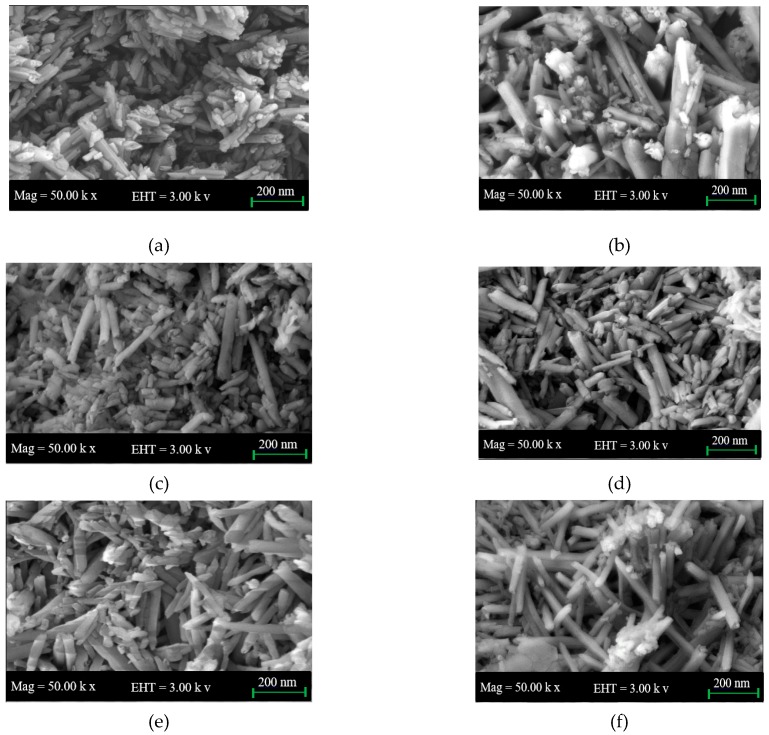
FESEM microphotographs for: (**a**) S01; (**b**) S02; (**c**) S03; (**d**) S04; (**e**) S05; and (**f**) S06.

Figure 4 shows the FESEM images of S01, S02, S03, S04, S05, and S06. The focus in these images is the distribution of the nanotubes before and after treatment HNTs with H_3_PO_4_. With the capability of FESEM, the images show the bulk of samples in addition to the surface morphology. Furthermore, the images of the samples did not show clearly the exfoliation on the outer surface. FESEM images reveal the empty lumen and tubular structure of nanotubes with open-ended tubes. In Figure 4c–e, it can be observed that nanotubes are non-uniformly dispersed, with agglomeration and cluster formations. After HNTs are treated and fabricated, as in Figure 4b–e, the clusters of nanotubes are decreased and the HNTs particles are separated from each other. In Figure 4d,e, it can be observed that the nanotubes are improved, the distribution is better and the HNTs particles are separated from each other. This is due to the crosslinking with MA and the presence of dispersing agent SDS that made the distribution better and reduced agglomeration [45,46]. It shows substantial improvements in nanotubes dispersion and a small cluster of nanotubes can be seen. Apparently, in Figure 4f, nanotubes appeared to re-agglomerate with no exact definite physical features of cluster formation, even though the nanotubes are still intact. FESEM results for S06 are consistent with TEM images for the same sample that shows how it is exfoliated on the outer surface.

### 3.5. Brunauer–Emmett–Teller (BET)

**Figure 5:**
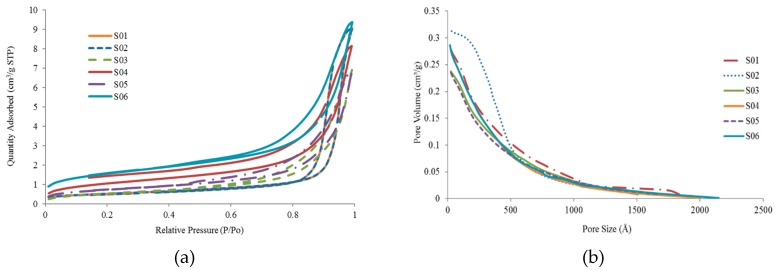
(**a**) N_2_ adsorption-desorption curves; (**b**) distribution of micropore size.

The N_2_ adsorption–desorption analysis is to investigate the surface area and pore volume of the nanotubes. Figure 5a shows the N_2_ adsorption-desorption curves for S01, S02, S03, S04, S05, and S06 while Figure 5b shows the distribution of micropore size of the same set of samples. The results of BET are listed in Table 6. HNTs can act as a door to one another by blocking the inner pore through aggregation or opening through dispersion [9,47]. From the adsorption and desorption curve, a small area is shown from the isotherm curves. This area is related to the number of N_2_ molecules adhering to the surface and not desorbed, and it becomes smaller as the HNTs are modified. After the modification of HNTs, there is a slight difference in the distribution of micropore size of HNTs (Figure 5b). In Table 6, the changes in BET surface area and the slight difference of total pore volume of the samples can be observed. As for S01, the BET surface area and total pore volume are 59.1 m^2^/g and 0.26 cm^2^/g, respectively. This result is consistent with the finding of Gaaz et al. [40]. There is a significant increase in BET surface area from 59.1 m^2^/g (S01) to 83.81 m^2^/g (S04) and 121.1 m^2^/g (S06). This is due to the high development of internal and external surface of HNTs [40]. However, the BET surface area decreases from 59.1 m^2^/g (S01) to 40.1 m^2^/g (S02) and 44.4 m^2^/g (S03), which are consistent with the reduction of total pore volume from 0.26 to 0.22 cm^2^/g. The decrease in the surface area suggests that the modification of HNTs succeed [48,49,50]. As the HNTs are modified with treated HNTs and addition of PVA, PVP, and crosslinker MA, BET surface increases to 121.13 m^2^/g (S06) while the average pore size decreases from 161.4 to 121.9 nm, suggesting that S06 had become less porous due to the crosslinking process [45]. It is observed in Table 6 that the results of the average pore size of S02 reflect significant increment from 167.37 to 304.60 nm, which could suggest that the number of pores increases, making it more porous than S01, as shown in Figure 5b [45].

### 3.6. Size Distribution

**Figure 6:**
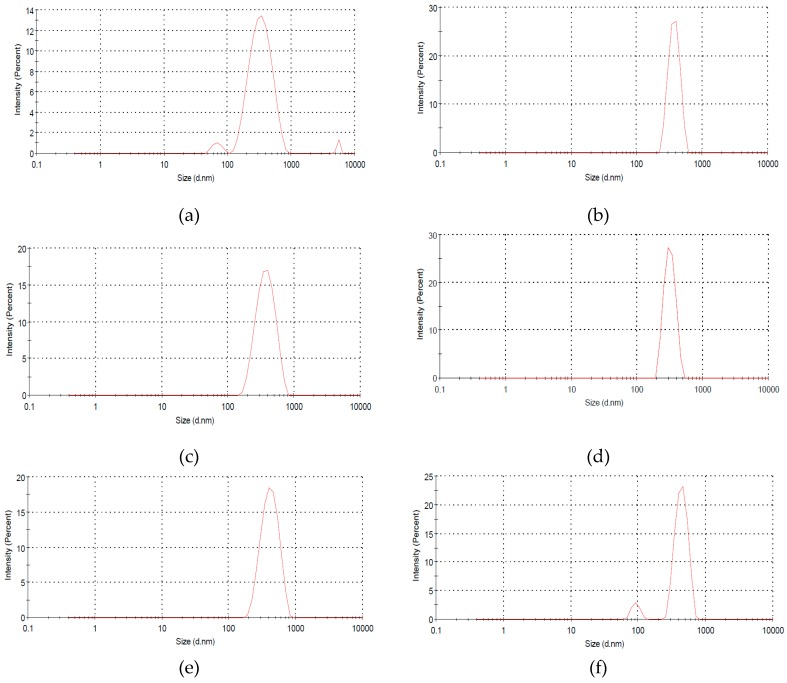
HNTs particle size distribution of: (**a**) S01; (**b**) S02; (**c**) S03; (**d**) S04; (**e**) S05; and (**f**) S06.

Particle size and distribution analysis are significant for physical stability and activity of colloidal system [51]. Figure 6 presents the particle size represented by the diameter of the particle and distribution for S01, S02, S03, S04, S05, and S06. In Figure 6a, the particle size (diameter) of S01 is 337.7 nm, as based on Liu et al. [52]; the size ranges of HNTs are 50 to 400 nm. It shows a significant variation in the particle size: 645.7, 398.2, 483.5, 493.4 and 588.2 nm for S02, S03, S04, S05, and S06, respectively. The increment of particle size is due to the modifications of HNTs [51]. Particle size larger than 400 nm, however, corresponds to agglomeration [53]. S06 has a particle size of 588.2 nm, which means it agglomerates, and this result is consistent with TEM images in Figure 3f. However, in Liu et al. [52], the particle size and distribution are practically independent of the composite of HNTs.

### 3.7. Atomic Force Microscopy (AFM)

**Figure 7:**
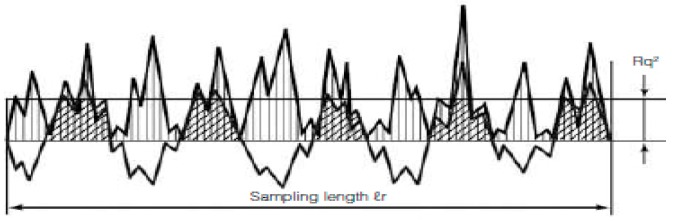
The surface appearance under AFM.

AFM analysis is carried out to study the surface topography or morphology and surface roughness of the samples. Figure 7 shows the AFM images for S01, S02, S03, S04, S05, and S06. The brightest area represents the highest point of the membrane surface while the dark region illustrates the valley or membrane pores [35]. The microscopic feature of the surface under AFM looks like the surface presented in Figure 7. The surface roughness listed in Table 7 represents the frequent heights of the real surface relative to troughs. The most important parameter for identifying the surface roughness is the root-mean-square height, as defined in Equation 1.

The Root-Mean-Square height:

(1) $$S_{q}=\sqrt{\frac{1}{A}\underset{A}{\iint }Z^{2}\left(x,\;y\right)dxdy}$$

The mathematical formula to measure the roughness.

**Figure 8:**
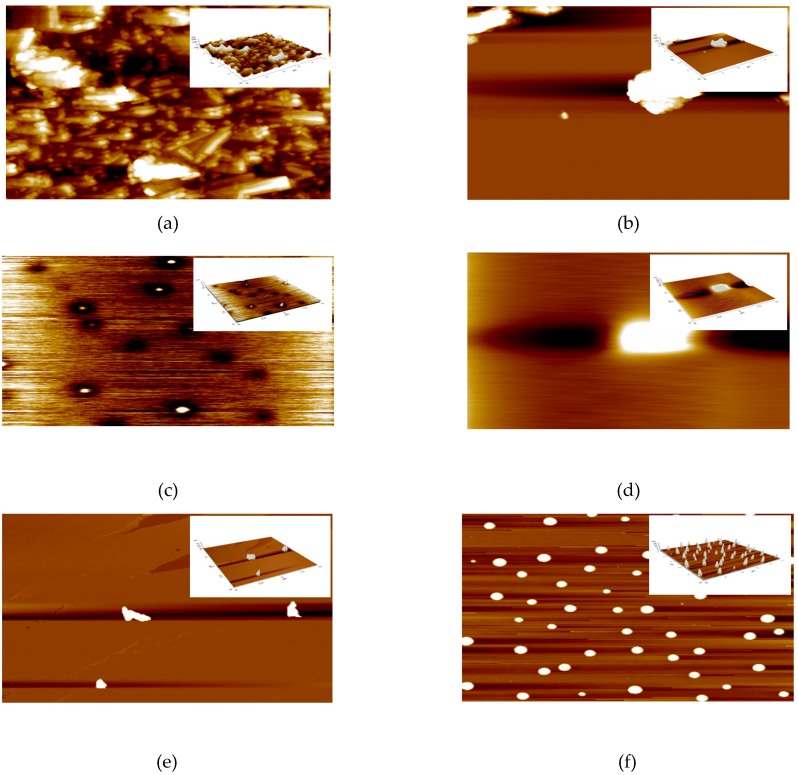
AFM 2D and 3D (insert) images of: (**a**) S01; (**b**) S02; (**c**) S03; (**d**) S04; (**e**) S05; and (**f**) S06.

As can be seen in Figure 8a, the surface of S01 shows it has “ridge-and-valley” texture with average roughness, $S_{q}$ of 41.4 nm. Before modification, S01 illustrates roughness surface texture while $S_{q}$ decreases after treating HNTs with H_3_PO_4_ to 24.6 nm, consistent with Figure 8b that illustrates the surface has less “ridges-and-valley” texture. Compared to S01, S02 is less aggregate. More rough surfaces have higher values of *S_q_* and vice versa [54]. After S01 is modified with the addition of PVA and PVP (Figure 8c), the *S_q_* significantly decreases from 41.4 to 0.4 nm. Less “ridge-and-valley” can be observed in Figure 8c. In contrast to S03, the *S_q_* of S05 slightly increases to 10.5 nm. This is due to the presence of the crosslinker MA in the composite [55]. From *S_q_* of 24.6 nm (S02), the *S_q_* of S04 decreases to 4.2 nm, while, with the addition of crosslinker MA (S06), it increases to 25 nm. As can be seen in Figure 8e (S04), the surface is smoother compared to Figure 8f (S06) that has more “ridge-and-valley” texture. This may be due to the re-agglomerate of S06, hence, confirming the TEM image in Figure 3f. It is observed that the surface area of modified HNTs decreases from S01 as reported by [54,56,57]. Smoothing of the composites is due to the good interaction and compatibility between nanotubes and polymer chains via hydrogen bonding and chemical crosslink by MA that further improve the compatibility [54,58,59]. Membrane fouling is a significant problem in membrane filtration, thus, it is important to reduce the surface roughness to improve the antifouling ability of the membrane [35].

## 4. Conclusions

HNTs–PVA–PVP composite has been investigated by numerous researchers due to their wide applications in industry and, more interestingly, in the medical field. The HNTs–PVA–PVP composite shows better characteristics regarding agglomeration when HNTs are treated in advance by H_3_PO_4_. In addition to this treatment, MA was used to crosslink PVA and PVP with HNTs. In this work, HNTs–PVA–PVP composite was prepared based on a unique procedure characterized by crosslinking two polymers with HNTs. The composite of two polymers fortified by the crosslinker MA and the dispersive agent SDS are prepared with neat and H_3_PO_4_ treated HNTs. FTIR, XRD, TEM, FESEM, BET, size distribution, and AFM are used to describe the properties, which show the effect of H_3_PO_4_ treatment and the cross-link. FTIR shows additional peaks at 2924.3, 1455.7, and 682.4 cm^−1^ compared to the neat HNTs due to adding MA. For XRD, even though the spectra do not show a significant difference, the decrease in peak intensity could be attributed to the addition of semi-crystalline PVA and the amorphous PVP. The images taken by TEM and FESEM show the possible effects of MA on the morphology and internal feature of HNTs–PVA–PVP composite treated by MA by showing the deformation of the matrix. The BET surface area increased to 121.1 m^2^/g compared to the neat HNTs at 59.1 m^2^/g. This result, the second highest recorded result, is considered a breakthrough for enhancing the properties of HNTs–PVA–PVP composite treated by MA crosslinking, and may be attributable to the size and number of pores. The results from these techniques clearly showed that a significant change has occurred for treated HNTs–PVA–PVP composite when MA was added. The characterization of HNTs–PVA–PVP composite with and without treating HNTs and using crosslinker may lead to a better understanding of this new composite as a precursor to possible applications in the dentistry field.

## Figures and Tables

**Table 1:** Chemical composition of HNTs.

Chemical Compositions	O:SiO_2_	Al:Al_2_O_3_	Si:SiO_2_
Weight%	61.19	18.11	20.11

**Table 2:** Physical properties of HNTs.

Chemical Formula	Surface Area	Pore Volume	Density
Al_2_Si_2_O_5_(OH)_4_·*n*H_2_O	60 m^2^/g	~1.25 mL/g	2540 kg/m^3^

**Table 3:** Other chemicals used in this work and their properties.

Materials	Typical Data	Value	Sources
SDS (C_12_H_25_NaO_4_S)	Molecular weight (g/gmol)	288.4	BioShop Canada Inc., Burlington, ON, Canada
	Melting point (°C)	204–20	
	pH	9.5	
	Density (g/cm^3^)	1.106	
PVA‎ (C_2_H_4_O)*_n_*	Molecular weight (g/gmol)	89–98	Sigma Aldrich, St. Louis, MI, USA
	pH	5–7	
	Viscosity (cpc)	11.6–15.4	
	Density (g/cm^3^)	1.269	
	Melting point (°C)	200	
PVP (C_6_H_9_NO)*_n_*	Molecular weight (g/gmol)	40	Sigma Aldrich, St. Louis, MI, USA
	pH	3–7	
	Viscosity	350–600	
	Density (g/cm^3^)	1.2	
	Melting point (°C)	150–180	
MA (C_3_H_4_O_4_)	Molecular weight (g/gmol)	104.06	Sigma Aldrich, St. Louis, MI, USA
	Purity (%)	98.5–101.5	

**Table 4:** Preparation of the six samples used in this paper.

Sample Name	Composition by Weight (g)				
	Neat HNTs	Treated HNTs	PVA	PVP	MA
S01	1.0 g	-	-	-	-
S02	-	1.0 g	-	-	-
S03	1.0 g	-	0.10 g	0.10 g	-
S04	-	1.0 g	0.10 g	0.10 g	
S05	1.0 g	-	0.10 g	0.10 g	0.10 g
S06	-	1.0 g	0.10 g	0.10 g	0.10 g

**Table 5:** Results of FTIR.

Sample		S01	S02	S03	S04	S05	S06
OH/O–H–Structure	O–H inner	3695.3	3694.8	3695.2	3695.4	3695.5	3695.9
	OH–inner	3624.3	3620.4	3627.1	5624.5	3626.7	3620.7
	O–H intramolecular	-	-	-	3430.2	-	-
C–H stretching and bending		-	-	2924.2		-	-
		2356.6	-	-	2357.3	-	-
		-	2104.1	-		2108.1	2114.8
		-	-	-		-	2011.5
[O–H]: deformation of [COOH] group		1650.5	1641.1	1652.5	1650.9	1650.9	1641.4
(C=O) mono disodium MA		-	-	-		1444.2	1442.2
Si–OH		1120.0	-	1121.2		1121.1	-
Si–O–Si		1033.3	1037.2	1030.6	1037.4	1031.5	1038.6
Al–OH		911.8	912.5	910.6	912.2	910.6	911.8
Al–O–OH		750.8	-	751.0		750.9	-
		-	796.4	-	795.3	-	795.5
		-	689.6	-	690.2	-	691.6

**Table 6:** Surface areas and pore volumes.

Sample	S01	S02	S03	S04	S05	S06
BET surface area (m^2^/g)	59.1	40.1	44.4	83.8	58.2	121.1
Total pore volume (cm^3^/g)	0.26	0.21	0.22	0.30	0.27	0.31
Micropore volume (cm^3^/g)	0.001	0.0001	0.0006	0.004	0.003	0.006
Mesopore volume (cm^3^/g)	68.0	41.3	54.4	83.7	61.1	105.3
Mesopore surface area (m^2^/g)	0.28	0.23	0.24	0.31	0.27	0.32
Average pore size (nm)	167.4	304.6	176.9	136.8	156.7	121.9

**Table 7:** AFM roughness measurements.

Sample	S01	S02	S03	S04	S05	S06
Roughness (*S_q_*) (nm)	41.4	24.6	0.4	4.2	10.5	25.0
